# Resistance Index of Penicillin-Resistant Bacteria to Various Physicochemical Agents

**DOI:** 10.5402/2012/789474

**Published:** 2012-01-31

**Authors:** M. Kazemi, R. Kasra Kermanshahi, E. Heshmat Dehkordi, F. Payami, M. Behjati

**Affiliations:** ^1^Department of Genetics and Molecular Biology, Medical School, Isfahan University of Medical Sciences, Isfahan 81746-73461, Iran; ^2^Department of Microbiology, Faculty of Science, The University of Isfahan, Isfahan, Iran

## Abstract

Widespread use of various antimicrobial agents resulted in the emergence of bacterial resistance. Mechanisms like direct efflux, formation, and sequestration of metals and drugs in complexes and antiporter pumps are some examples. This investigation aims to investigate the resistance pattern of penicillin-resistant bacterial strains to some physicochemical agents. Sensitivity/resistance pattern of common bacterial strains to antimicrobial agents were evaluated by disk diffusion assay. Broth and agar dilution method were used for determination of minimum inhibitory concentration and minimal bactericidal concentration. The impact of UV ray on the bacterial growth under laminar flow hood was measured using photonmeter. Our data demonstrates that the most prevalent metal resistance was against arsenate (95.92%), followed by cadmium (52.04%) and mercury (36.73%). There was significant difference between cetrimide resistances among studied microbial strains especially for *P. aeruginosa* (*P* < 0.05). High rate of pathogen resistance to various antibacterial agents in our study supports previously published data. This great rate of bacterial resistance is attributed to the emergence of defense mechanisms developed in pathogens. The higher general bacterial resistance rate among *Staphylococcus* strains rather than *E. coli* and *P. aeruginosa* strains draws attention towards focusing on designing newer therapeutic compounds for *Staphylococcus* strains.

## 1. Introduction

Various resistance mechanisms have been developed by bacteria to counteract heavy metal stress. Some of these mechanisms include metal efflux out of the cell, sequestration of heavy metals in complexes, and reduction of a metal to less toxic species [1, 2]. Metal efflux is seen in the case of Cd which is detoxified in gram-negative bacteria by RND-driven system like Czc and Ncc as zinc and Nickel exporters, respectively [3]. In gram-positive bacteria this metal efflux is done via Cadmium-exporting *P*-type ATPase as seen in CadA pump in *S. aureus*. Hg^2+^ is transported inside the cells by specific uptake system which is rapidly reduced to abolish the toxic effects of Hg^2+^ on periplasmic proteins by reoxidation. In this way, Mercury leaves the cell by passive diffusion and does not remain inside the cell [4, 5]. The first detoxification step in gram-negative bacteria is attachment of periplasmic Hg^2+^-binding protein MerP to cations. It probably delivers toxic cations to the mercury transporter system which is subsequently transported into the cytoplasm. An alternative uptake route is also present which involves MerC protein. Inside the cell, Hg^2+^ is reduced into Hg^0^ by MerA, related to the glutathione reductase system. MerA protein is also involved in the reduction process of Hg^2+^ after cleavage by MerA. MerB detoxifies organomercurial agents which are much more toxic than Hg^2+^ [5].

Multidrug resistance pumps (MDR), responsible for the extrusion of chemically unrelated antimicrobial agents composes alternative resistance pathway. This process is mediated by active export of the toxic compounds by means of the proton motive forces [6]. Multidrug resistance to various organic cationic antiseptics and disinfectant compounds as cetyltrimethylammonium bromide (cetrimide) has been reported in various organisms including *S. aureus, E. coli*, and *B. subtilis* [7, 8]. Mutations leading to overexpression of the pumps have been identified in clinical isolates of multidrug-resistant strains [9]. Among these strains, *P. aeruginosa* is an opportunistic human pathogen with innate resistance to multiple antimicrobial agents. This intrinsic multidrug resistance is attributed to the lowpermeability of outer membrane and expression of a number of broadlyspecific multidrug efflux (Mex) pumps as MexAB-OprM and MexXY-OprM [2, 9]. These pumps are composed of three different peptides: MexA, a fusion membrane protein which apparently docks MexB to OprM, MexB translocase which belongs to the resistance nodulation division (RND) family of solute/proton antiporters and OprM which is an outer membrane porin [10]. The broad specificity of MDRs seems to be matched with the broad resistance of biofilms to antimicrobial agents. MDRs are mostly regulated by environmental factors exemplified by induced expression of *E. coli *EmrAB and RND pump by drug substrate and stress, respectively [10]. The presence of three additional efflux systems as MexCD-OprJ, MexEF-OprN, and MexJK-OprM due to overexpression of efflux genes by mutational events is addressed by other studies. However these pumps play different roles rather than drug efflux including export of the biocides, dyes, detergents, metabolic inhibitors, organic solvents, and molecules participated in bacterial cell-to-cell communication [11]. In *P. aeruginosa*, the MexAB-OprM multidrug efflux system exports a number of antimicrobial compounds such as *β*-Lactams and is responsible for its “intrinsic resistance” to antibiotics. The substrates for MexAB-OprM include quinolones, tetracycline, and *β*-lactams [2, 9, 12].

UV light is recognized as an effective antiorganism by inactivation of pathogens [13]. The effectiveness of UV light in biological inactivation aroused from observation of double stranded DNA breaks occurred by UV ray [14]. The break point is particularly between pyrimidine bases which alters base pairing and induces formation of new linkage between adjacent nucleotides on the same DNA strand. Unrepaired damage blocks DNA replication which ultimately lead to cell cycle arrest and death [14]. Many organisms developed reparative mechanisms to compensate destructive effects of UV radiation as nucleotide excision repair and photo reactivation [11]. Since UV radiation is widely used to sterilize operation rooms, utensils, and drinking water, the emergence of bacterial resistance will cause great hygiene problems.

As mentioned above, various drug resistance mechanisms are involved in the emergence of pathogen-drug resistance. It is not unusual for pathogens to use combination of these resistance strategies against antimicrobial agents. Understanding the synergistic effects of these resistance strategies helps us choose appropriate therapeutic agents against developed resistance. Therefore, the aim of this study is to evaluate the prevalence of simultaneous resistance to the above-mentioned factors in some common resistant pathogens.

## 2. Materials and Methods

### 2.1. Bacterial Strains

Standard bacterial strains were cultured in commercial culture medias as Nutrient agar (NA), Nutrient broth (NB), Trypticase-soy agar (TSA), Trypticase-soy broth (TSB), Muller Hinton agar (MHA), Eosin methylene blue (EMB), and MacConkey agar. PHG-II and biochemical culture medias were also used for heavy metal resistance and identification of isolated bacteria, respectively. Cadmium nitrate, sodium arsenate, and mercury nitrate were used as heavy metals in this study (Table 1).

### 2.2. Determination of Bacterial Sensitivity to Antimicrobial Agents

In order to determine the sensitivity/resistance pattern of bacterial strains to antimicrobial agents, disk diffusion assay (Kirby-Bauer method) was used. Broth and agar dilution method was used for determination of minimum inhibitory concentration (MIC) and minimal bactericidal concentration (MBC). Determination of the optimal concentration of antimicrobial compounds with the capacity to annihilate 99.9% of the microorganisms was performed using agar on plate method. Bacterial resistance to cetrimide was assessed by replica-plating method using steer's replicator [15, 16]. Sterile PHG-II medium supplemented with the Peptone (4 g/L), yeast extract (1 g/L), Glucose (2 g/L), and Agar (15 g/L) was used to determine MIC against heavy metals. Various concentrations of heavy metals added to this medium in plates (adjusted pH and 55°C).Then, 0.1 mL of microbial suspension in Log-phase was spread on plates. Subsequently, plates were incubated at 35°C for 24–72 h [17, 18]. Different concentrations of heavy metals were used as follow:

- cadmium nitrate: 0.037–0.075–0.15–0.31–0.62–1.23–2.46–4.93 (*μ*g/mL),
- mercury nitrate: 0.12–0.25–0.50–1.00–2.00–4.00 (*μ*g/mL),
- sodium arsenate: 2–4–8–16–32–64–128 (mg/mL).

Resistance strains to chemical agents were based on the following relations:

- cetrimide [19],
- in *S. aureus* and *E. coli*, respectively, growth at the concentrations of 4.16 and 20.83 *μ*g/mL in screening method and* P. aeruginosa*, MIC ≥ 400 *μ*g/mL,
- metals [17, 19],
- cadmium, MIC ≥ 0.62 *μ*g/mL,
- mercury, MIC ≥ 1 *μ*g/mL,
- arsenic, MIC ≥ 4 mg/mL.

### 2.3. Determination of Bacterial Sensitivity to UV Radiation

The impact of UV ray (UVC) on the bacterial growth under laminar flow hood (Slee Mains VLFS 636) was measured using photonmeter (Hausatech Quantum sensor Q SPAR). Grew bacteria in TBS (24 h, 30 rpm) were diluted in order to achieve approximately 1.5 × 10^8^/mL Bacteria. Then, 0.1 mL of the solution was spread evenly on TSA plates. Thereafter, bacterial cultures were exposed to UV radiation in different time intervals (0, 30, 60, 120, and 240 Sec) at the intensity of 0.25 j/m^2^s. All samples were incubated at 35°C for 4 h before scoring. The coefficiencies of sensitivity to UV radiation were determined using the following formula: S_UV_ = Ln [(CFU) d/(CFU) 0]/d where (CFU) 0 indicates the number of bacteria in the certain volume of control samples before UV radiation, (CFU) d indicates the number of bacteria in the same volume of sample after UV radiation, and d stands for applied dose in terms of j/m^2^ [15, 20].

### 2.4. Statistical Analysis

Statistical analysis between different experimental assays was performed by SAS software using general linear models and correlation analysis procedures. All data are expressed as Mean ± SD. *P* values less than 0.05 were accepted as statistically significant difference.

## 3. Results and Discussion

**Figure 1:**
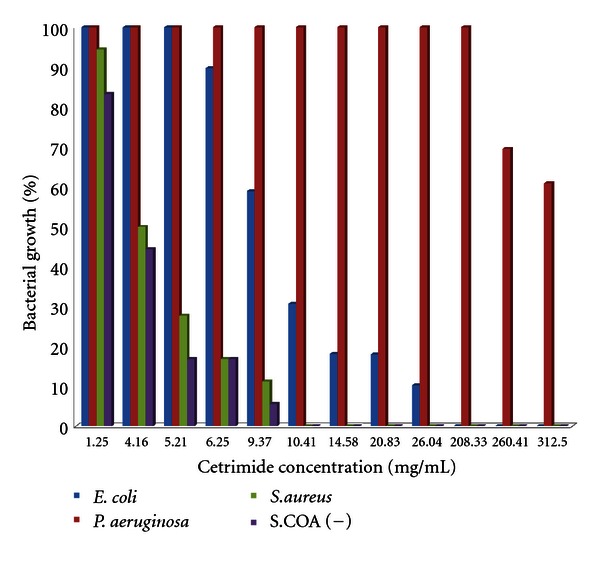
Comparison of bacterial growth rate in the presence of various concentration of cetrimide.

The percentage of bacterial growth in the presence of different concentrations of cetrimide is demonstrated in Figure 1. A considerable difference in resistance to cetrimide has been observed between microbial strains. The greatest resistance rate to cetrimide was imparted by *P. aeruginosa*. The greatest MIC and MBC values were 6.25 *μ*g/mL and 25 *μ*g/mL, 50 *μ*g/mL and 200 *μ*g/mL, and 400 *μ*g/mL and 800 *μ*g/mL for *Staphylococcus, E. coli*,* and P. aeruginosa*, respectively. A significant difference (*P* < 0.05) was observed between MIC and MBC of *Staphylococcus and E. coli with P. aeruginosa* strains.

In general, the most prevalent metal resistance was against Arsenate (95.92%), followed by Cadmium (52.04%) and Mercury (36.73%) (Table 2). The rate of double metal resistance was 25.51%, 52.04%, and 36.67% for Cd-Hg, Cd-As and Hg-As, respectively. The rate of triple metal resistance (Cd-Hg-As) was 25.51.

**Figure 2:**
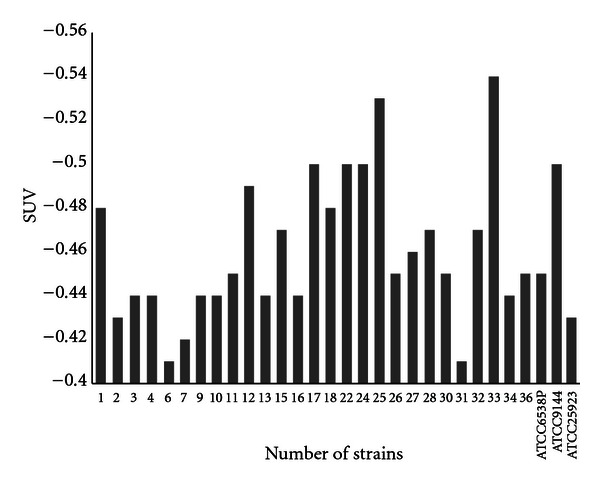
Comparison of coefficient of sensitivity to UV radiation (S_UV_) in *S. aureus* strains.

**Figure 3:**
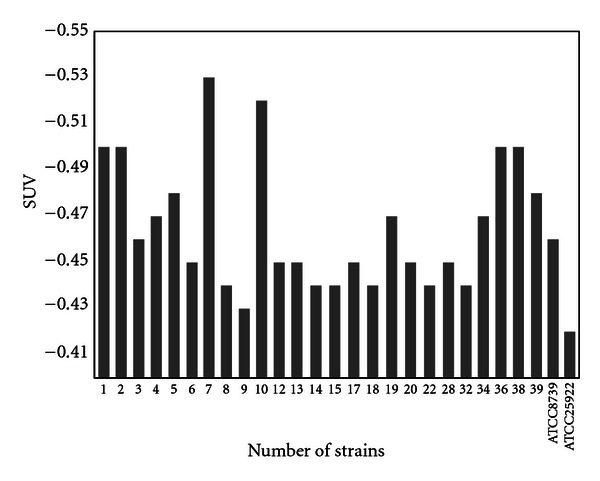
Comparison of coefficient of sensitivity to UV radiation (S_UV_) in *E. coli* strains.

**Figure 4:**
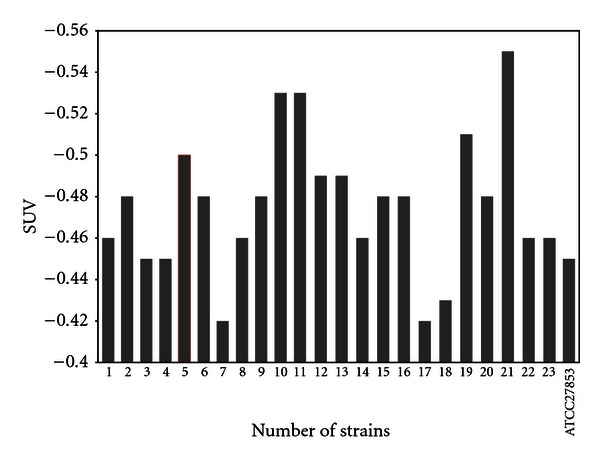
Comparison of coefficient of sensitivity to UV radiation (S_UV_) in *P. aeruginosa* strains.

**Figure 5:**
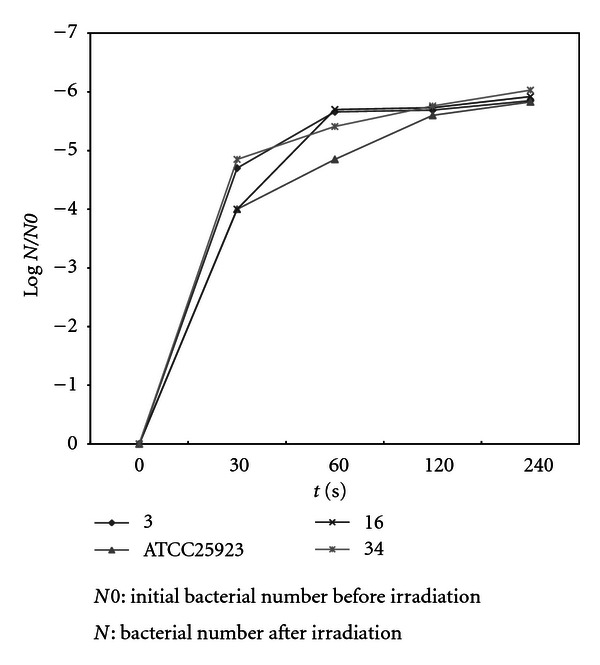
Comparison of survival rates before and after UV irradiation (0.25 J/m^2^S) in *S. aureus* strains.

**Figure 6:**
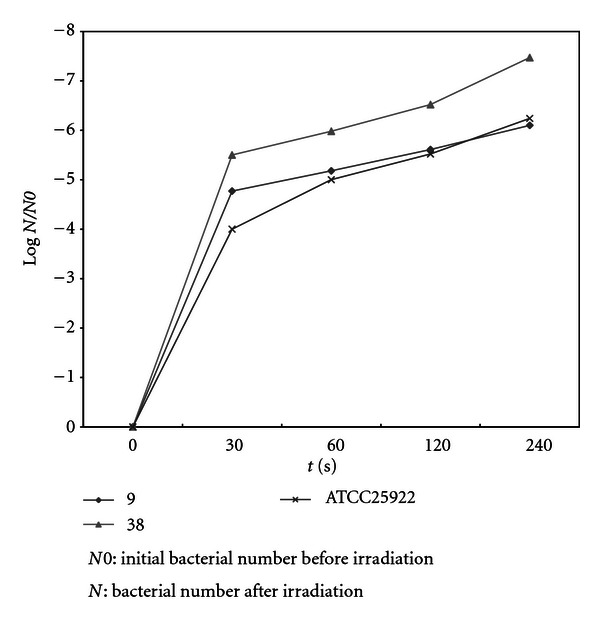
Comparison of survival rates before and after UV irradiation (0.25 J/m^2^S) in *E. coli* strains.

**Figure 7:**
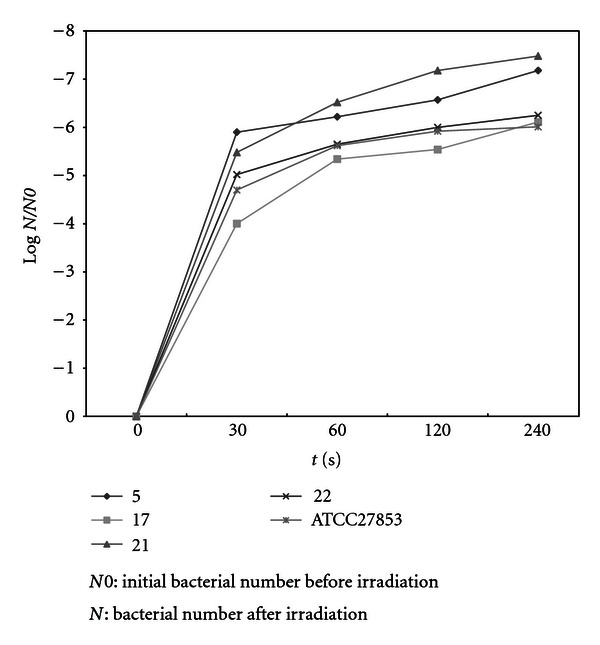
Comparison of survival rates before and after UV irradiation (0.25 J/m^2^S) in *P. aeruginosa* strains.

Comparison of coefficient of sensitivity to UV rays for the strains of *Staphylococcus, E. coli*, and *P. aeruginosa, *are demonstrated in Figures 2–4. In order to evaluate bacterial resistance to UV ray, heavy metals and cetrimide-resistant strains were exposed to UV ray at intensity of 0.25 J/m^2^s for 0, 30, 60, and 120 and 240 second time spans (Figures 5, 6, and 7). Mean colony numbers grown in the culture medium after 240 seconds of UV radiation were 3.6, 4.7, and 6.9 in the strains of *P. aeruginosa*, *E. coli*,* and *Staphylococci, respectively. Mean numbers of the colonies after 120 seconds of UV irradiation were, respectively, 21.8, 16.2, and 13.5 in the strains of *Staphylococcus*, *E. coli* and *P. aeruginosa. *There was a significant difference between *Staphylococcus *and standard strains in each group after 60 seconds. Such difference was not observed between standard strains, *E. coli,* and *P. aeruginosa.* There was no significant difference between examined strains after 120 seconds. After 240 seconds, there was a significant difference between *Staphylococcus* and *P. aeruginosa* but not with *E. coli*. Our data demonstrates that the *Staphylococcus*, *E. coli* and *P. aeruginosa* have mean sensitivity coefficient to UV ray (S_UV_) of −0.461, −0.466, and –0.476, respectively, (Figures 3–5). The mean colony number in all the strains under study is equal to 52.40, 17.68, and 5.46 in 60-, 120-, and 140- second time spans for *Staphylococcus*, *E. coli,* and *P. aeruginosa*, respectively. Thus, the difference between the colony numbers in 60, 120, and 240 seconds of UV radiation was not significant in all of the studied strains (*P* > 0.05, CI of 95%).

Presence of multidrug resistance pumps with capability to extrude chemically unrelated antimicrobial agents has been demonstrated in various living organisms. Indeed, gram-negative bacteria exhibit low susceptibility to many antibiotics like penicillin, compared with gram-positive strains [21]. It means ineffectiveness of its most effective gram-positive antibiotics for gram-negative bacteria. Intrinsic resistance of gram-negative bacteria is mainly related to the presence of outer membrane barriers. These barriers act as narrow porin channels, which slows down penetration of even small hydrophilic solutes and low fluidity of lipopolysaccharide. These channels decrease the rate of transmembrane diffusion of lipophilic solutes. There are some clues regarding the presence of other mechanisms besides outer membrane barriers even with species such as *P. aeruginosa*, which produce an outer membrane of exceptionally low permeability [22]. Rapid gain of equilibration across the outer membrane is partly attributed to the large surface-to-volume ratio in a small bacterial cell. Therefore, the periplasmic concentrations of many antibiotics are expected to reach 50% of their external concentrations in 10 to 30 seconds in *P. aeruginosa* and in a much shorter time period than *E. coli*. Additional mechanisms are therefore needed to explain intrinsic resistance like the hydrolysis of the earlier *β*-lactam compounds by the periplasmic *β*-lactamases encoded by chromosomal genes in many gram-negative bacteria [23]. However, recent studies showed that multiple drug efflux pumps with broad specificities play a major role in intrinsic resistance of gram-negative bacteria [24].

Cetrimide, used widely as biocide and disinfectants, entered in the pathogen cell wall via outer membrane permeability porins. Cetrimide resistance phenotype encoded by plasmid genes in *E. coli *was shown to be associated with altered composition of outer membrane lipopolysaccharide and diminished porin numbers. Cetrimide resistance has been shown with *Staphylococcus*, *E. coli*, and *P. aeruginosa*, similar to our findings. The greatest cetrimide resistance by *P. aeruginosa* seen in our study supports the finding regarding great gram-negative bacteria, mainly *Pseudomonas, *resistance to cetrimide and multiple antibiotics, especially during nosocomial infections. Generally, gram-negative bacteria are more biocide resistant than gram-positive bacteria, but it is not always the case [25]. Pattern of heavy metal resistance in our studied isolates remark the relative effectiveness of mercury rather than cadmium and specially arsenate for these isolates. Great bacterial resistance against arsenate and cadmium makes them poor antimicrobial agents for these organisms, but new agents composed of mercury can be applied more effectively. Interestingly, our data demonstrates that double (mainly with mercury and cadmium) or triple metal usage might be more effective because of the lower bacterial resistance to these heavy metals. Application of ultraviolet spectrums, germicidal (ultraviolet C) and solar (ultraviolet A and B), as adjunctive therapy for bioburdens have been previously documented [20]. In our study, UV-radiation resistance in *Staphylococcus* strains was higher than that of the other groups and the strains of *E. coli* have a higher resistance than *P. aeruginosa.*

Overall, our data demonstrates that the general bacterial resistance rate was higher among *Staphylococcus* strains than *E. coli* strains. The same ranking was seen with *E. coli* strains comparing with *P. aeruginosa* strains. Despite of the previously published data, regarding the greatest rate of multiple antimicrobial resistances in gram-negative bacteria, our results demonstrated greater general bacterial resistance in *Staphylococcus* strains, a gram-positive bacterium. Knowing the great antimicrobial resistance rate in gram-negative bacteria, it will be easy to understand the meaning of the greater rate of resistance among *Staphylococcus* strains. These findings imply the very limited remained therapeutic options and consequently the need for finding new powerful antimicrobial agents.

## Figures and Tables

**Table 1:** Standard bacterial strains applied in this study.

Group	Strain	Application of strains
*S. aureus*	ATCC 6538P	Measurement of antimicrobial material
	ATCC 9144	Measurement of materials such as Cetrimide erythromycin, Penicillin and tetracycline
	ATCC 25923	Study of resistance against UV ray

*E. coli*	ATCC 8739	Measurement of antimicrobial materials
	ATCC 25922	Study of resistance against UV ray

*P. aeruginosa*	ATCC 9027	Measurement of antimicrobial materials
	ATCC 27853	Study of resistance against UV ray

**Table 2:** Bacterial resistance pattern to heavy metals.

Group	Number of strains	Bacterial resistance (%)						
		Cd	Hg	As	Cd-Hg	Cd-As	Hg-As	Cd-Hg-As
*S. aureus*	18	2* (11.11)**	7 (38.89)	14 (77.78)	0 (0)	2 (11.11)	4 (22.22)	0 (0)
SCN	18	8 (44.45)	0 (0)	18 (100)	0 (0)	8 (44.45)	0 (0)	0 (0)
*E. coli*	39	18 (46.15)	10 (25.64)	39 (100)	6 (15.38)	18 (46.15)	10 (25.64)	6 (15.38)
*P. aeruginosa*	23	23 (100)	19 (82.61)	23 (100)	19 (82.61)	23 (100)	19 (82.61)	19 (82.61)
Total	98	51 (52.04)	36 (36.73)	94 (95.92)	25 (25.51)	51 (52.04)	33 (33.67)	25 (25.51)

*Number of evaluated strains for each compound.

**The prevalence of bacterial resistance rate (%).
